# Immunological decoding of adult T cell leukemia – its cell of origin and oncogenesis

**DOI:** 10.1186/s40364-026-00968-2

**Published:** 2026-07-02

**Authors:** Toshiyuki Hori

**Affiliations:** Research Organization of Science and Technology, Ritsumeikan University, 1-1-1 Nojihigashi, Kusatsu, Shiga 525-8577 Japan

**Keywords:** ATL, HTLV-1, Naïve T cells, Tscm, Neonatal T cells, Quiescence, Niche, Oncogenesis

## Abstract

Adult T cell leukemia (ATL) is defined as a mature T cell neoplasm caused by human T cell leukemia virus type 1 (HTLV-1) and has been ascribed to the HTLV-1 gene products. However, the presence of asymptomatic carriers (ACs) accounting for the majority of HTLV-1 infected individuals who pass entire lives without developing ATL suggests that HTLV-1 infection alone is not sufficient for the onset of ATL. Studies with chronologically collected specimens from ATL patients or pre-onset individuals reported the presence of HTLV-1 infected CD4^+^ CD7^+^ CD45RA^+^ T cells in blood in HTLV-1 ACs and the step wise development of malignant clones from CD7^+^ T cells, indicating that ATL is caused by accumulation of driver gene mutations as other cancers in general. Epidemiologically, mother-to-child infection of HTLV-1 during neonatal period is known to be the most important determinant of overall ATL incidence. This observation has attracted attention as getting to the core of the matter but has lacked hitherto any appropriate reasoning. Recent progress in basic immunology has disclosed that neonatal naïve T cells are, distinct from adult naïve T cells, exceptionally long-living and have propensity toward regulatory T cells. A minor population of stem cell like memory T cell (Tscm)-like HTLV-1 infected cells were detected in ATL patients and reported to repopulate ATL clones in immunodeficient mice. In this context, we need to trace the origin of ATL to HTLV-1 infected CD7^+^ T cells or Tscm in blood in ACs, and further to HTLV-1 infected neonatal naïve T cells. Latent infection of HTLV-1 has long-term effects on naïve T cells not only by viral gene products but also through their own pattern-recognition receptor-mediated reactions. In sum, the distinctive feature of ATL lies in its origin which may involve a neonatal naïve T cell and persistence of HTLV-1 provirus. The latter facilitates driver gene mutations when the infected cell increases frequency of cell division in the middle age of life.

## Background

The oncogenesis of adult T cell leukemia (ATL) is still an enigma. While human T cell leukemia virus type 1 (HTLV-1) is recognized as the causative agent of ATL, there remain several fundamental questions: (1) why a small proportion of HTLV-1 carriers develop ATL after decades of latent period, (2) why mother-to-child infection of HTLV-1 during neonatal period is the most important determinant of overall ATL incidence, (3) why the incidence of ATL increases sharply in the middle age, (4) how HTLV-1 infected T cells persist in most asymptomatic carriers (ACs) without overt inflammatory or malignant diseases, (5) how HTLV-1 is involved in ATL oncogenesis at all.

Conventionally, the oncogenesis of ATL has been explained on the preconceived notion that it is caused by HTLV-1 viral factors and the origin of ATL has simply been thought to be HTLV-1-infected regulatory T (Treg) cells because of the phenotypical resemblance. I think that these explanations have lacked insights into the developmental biology of human T cells after exit from the thymus, especially the distinctive nature of neonatal naïve T cells.

In the present review, I briefly summarize the history of ATL research carried out on the premise of the hypothetical viral oncogenesis. After that, to answer the above-mentioned questions, I present a conceivable scenario of ATL development based on the recent research findings from the viewpoint of developmental biology of human T cells, and suggest future directions for treatment and prevention of ATL.

### Concept of adult T cell leukemia

ATL was proposed by K. Takatsuki and his collaborators as a distinct disease entity in 1976, based on age of onset, clinical symptoms, characteristic morphology of leukemic cells, and patients’ birthplace in Japan [1]. In this original article, the authors wrote, “Genetic background may play an important role but other factors such as oncogenic virus infections must be explored”, referring to the peculiar geographical distribution.

In 1980, a novel human retrovirus was isolated from a patient with cutaneous T cell lymphoma in the United States and named as “human T cell leukemia virus type 1” (HTLV-1) by R. Gallo and his collaborators [2]. Independent of this study, a retrovirus was detected in T cell lines derived from Japanese ATL patients in 1981 and further characterized as ATL-associated virus (ATLV) [3, 4]. After information exchange at an international workshop held in Kyoto in 1981, ATLV was recognized as identical to HTLV-1 [5, 6]. Antibodies against HTLV-1 antigens were present in sera of ATL patients [7] and monoclonal integration of HTLV-1 provirus was demonstrated in primary ATL samples examined [8]. With these findings taken together, ATL was redefined as peripheral T cell leukemia/lymphoma associated HTLV-1 infection [9]. In the Revised European-American Classification of Lymphoid Neoplasms published in 1994, ATL was defined as a T-cell neoplasm caused by HTLV-1 [10]. This etiological concept of the disease was taken over to WHO classification in 1997 and remained unchanged throughout revisions until the last 5th edition [11, 12]. Ever since, all the clinical, pathophysiological and epidemiological studies regarding ATL have been carried out on the premise of the hypothetical viral oncogenesis.

However, the relationship between ATL and HTLV-1 was not so simple as initially assumed, because it became clear that there were a large number of HTLV-1 ACs. According to a survey in 2012, there are estimated 5–10 millions of HTLV-1 carriers around the world, mainly in the endemic regions [13], but only 5–10% of them develop ATL [14]. While heterogeneity has been recognized among carriers with regard to proviral load and some possible subclinical effects [15], more than 90% of them anyhow pass their entire lives without developing ATL. Furthermore, distinction of a subset of ATL from other T cell neoplasms using clinical presentation, morphology and immunophenotype is often difficult if not impossible [16]. There are T cell leukemia/lymphoma cases without HTLV-1 proviral integration yet quite similar to typical ATL [17, 18]. It is also noted that T cell neoplasms other than ATL can develop in HTLV-1 infected individuals [19, 20]. On the other hand, rare cases of HTLV-1-positive (monoclonally integrated) cases of apparent angioimmunoblastic T cell lymphoma [21], mycosis fungoides [22], anaplastic large cell lymphoma [23], CD4^−^ CD8^+^ T cell lymphoma [24] and even Hodgkin lymphoma [25] have been reported. These cases are likely to have respective oncogenesis different from typical ATL. Thus, the etiological concept of ATL might be practically flawed.

### Roles of virus gene products

Like Epstein-Barr virus (EBV), HTLV-1 can transform normal lymphocytes. T cells are activated by HTLV-1 infection and induced to proliferate in vitro, occasionally giving rise to immortalized cell lines. This is one of the reasons why the oncogenicity of HTLV-1 has been intensively investigated [4]. HTLV-1 expresses multiple gene products encoded in both strands of the viral genome with mRNA splicing. In addition to the typical *gag*,* pro*,* pol*,* and env* genes, several overlapping reading frames were recognized in a region called pX flanked by two long terminal repeats (LTRs) [26]. These transcripts correspond to REX, Tax, small accessory proteins, and HTLV-1 bZIP factor (HBZ) which was identified years later [27, 28]. For more than 40 years since the discovery of HTLV-1, the studies on the viral oncogenesis were focused on the two viral proteins Tax and HBZ.

Tax activates NF-κB and upregulates expression of not only viral genes but also cellular genes that mediate cell proliferation [29–31] although expression of Tax can cause DNA damage and cellular senescence [32, 33]. Various types of *tax* transgenic mice models were made and the one using the *lck* promotors developed lymphomas after prolonged latent periods [34]. However, Tax is the major target of cytotoxic T cells (CTL) [35] and is not expressed in about half of ATL patients due to nonsense mutation, deletion of *tax* gene or DNA methylation in of 5’-LTR [36–40], suggesting that Tax is not indispensable at least in the final stage of ATL development.

Another viral gene product HBZ is not only invariably conserved and expressed in most ATL tumor cells but also counteracts Tax-mediated viral gene transcription to keep latent infection state [41, 42]. HBZ promotes T cell proliferation in its RNA form, whereas HBZ protein suppresses Tax-mediated viral transcription through the 5 L [43]. Transfection of *HBZ* induces expression of FOXP3 and BAFTF3 in T cells, leading to differentiation into Treg like cells and cell growth [44]. *HBZ* transgenic mice using the CD4-specific promoter/enhancer/silencer were made and some of them developed T cell lymphoma after long latent period with low penetrance [45]. In 2015, a gain-of-function mutation of CARD11 in the linker domain (CARD11^E626K^) was detected in ATL patients [46]. Taking this into account, double transgenic mice expressing CD4^+^ T cell-specific CARD11^E626K^ and/or CD4^+^ T cell-specific HBZ were made and most of them developed lymphoma after prolonged latent periods [47].

As anticipated since its discovery in an ATL-derived cell line, the viral factors of HTLV-1 were shown to induce T cell neoplasms in the mouse model systems, but with a caveat. It is a matter of debate whether Koch’s postulates can be applicable in the case like this [48]. The long latent period before the onset of lymphomas and the imperfect penetrance left the precise oncogenic process as a black box.

### Comparison with other types of PTCL

ATL belongs to mature T cell neoplasms which are quite diverse, ranging from a wide variety of peripheral T cell lymphomas (PTCL) to a few rare types of leukemias such as Sézary syndrome and T chronic lymphocyte leukemia/T prolymphocytic leukemia (T-PLL) [49]. The classification has been based on morphology, immunophenotype, genotype, and clinical features.

The diversity seems to reflect the complicated cell fate of T cells after exit from the thymus [50]. Unlike other hematopoietic cells, lymphocytes, especially T cells, undergo epigenetic reprograming to become naïve T cells which are self-renewing genuine stem cells. Naïve T cells continuously recirculate between secondary lymphoid organs, lymph and blood. In lymph nodes or spleen, they repeat active but slow self-renewal in niche in a state named as quiescence [51]. Some of them spontaneously converted to memory phenotype T cells or stem cell-like memory T cells (Tscm) through homeostatic proliferation elicited by contact with self-ligands [52, 53]. Upon exposure to exogenous cognate antigen presented by antigen presenting cells, naïve T cells are activated to become effector T cells which migrate to sites of the lesion to facilitate removal of pathogen. Most effector CD4^+^ T cells are short-lived, but a proportion survive as long-lived memory T cells or Treg cells which play important roles in maintaining and adjusting long term immunity [54]. Evidence has indicated that naïve CD4^+^ T cells contain inherent Treg cells distinct from effector memory type Treg cells [55]. Since both naïve T cells and memory T cells are long-lived and have potential of self-renewal, it seems reasonable to speculate that the origin of each mature T cell neoplasm is the corresponding type of long-lived T cells.

Recent progress in sequencing technology has disclosed the genetic landscapes of various types of PTCL and ATL. As for the cell type classification, gene expression profiling by quantification of specific transcripts from formalin-fixed paraffin-embedded (FFPE) samples demonstrated an association between the pathological subtypes of PTCL and the major helper T cell (Th) subsets [56–58]. While most nodular types of PTCL were assigned to either effector Th subsets as the cell of origin, the transcriptomic profile of ATL was not similar to that of any of them except for Treg cells, aside from the presence of the viral gene transcripts. These results might support the repeated claim that ATL derives from Treg cells [59–62]. In fact, it is well known that the majority of ATL cells have the phenotype of CD45RO^+^, CD7^−^, CD25^+^, Foxp3^+^ which corresponds to conventional effector Treg cells. However, ATL cells have been demonstrated to be functionally different from effector Treg cells [63, 64]. In addition, the phenotypes of HTLV-1 infected cells and ATL cells can vary across individuals and disease states [65].

Then, which type of cell is the origin of ATL? Is it an unknown type of effector memory T cells? We can ask this question in several different ways, but the point is how HTLV-1 infected cells remain for a long period of time without causing any particular disorders in ACs.

### Characterization of HTLV-1 infected cells in ACs

In 2014, S. Kobayashi et al. carried out CADM1 versus CD7 multi-color analysis of CD4^+^ T cells in peripheral blood mononuclear cells from ATL patients and ACs [66], based on the preceding finding that CADM1 is expressed on most HTLV-1 infected cells [67, 68]. The results were summarized into; (1) HTLV-I–infected and clonally expanded cells were efficiently enriched in CADM1^þright^ subpopulations, (2) the proportions of the three subpopulations in the CADM1 versus CD7 plot, discriminated by CADM1 expression and stepwise downregulation of CD7, accurately reflected the disease stage in HTLV-I infection, and (3) the CADM1^þright^ CD7^dim/negative^ subpopulations were at the intermediate stage of ATL progression and could be identified even in ACs. To be exact, expression of CADM1 is not specific for HTLV-1 infected cells [69] and is related to NF-κB activation [70]. However, it has been confirmed that CADM1 is the best single marker of HTLV-1 infection, identifying HTLV-1-infected cells with greater sensitivity and specificity than CD25, CCR4 or ICAM-1 [71]. Going back to the last question, the stepwise downregulation of CD7 from high to low levels in CADM1^+^ cells suggests that most HTLV-1 infected cells used to be CD7^high^ T cells before the onset of ATL. These CD7^high^ cells do not seem to have experienced exogenous cognate antigen stimulation because effector T cells including Treg cells are basically CD7^−^ or CD7^low^.

In 2021, an integrated study of genome sequencing and single-cell simultaneous transcriptome with cell surface protein analysis was carried out using chronologically collected specimens from patients with ATL or pre-onset individuals, indicating that multistep mutations in the T-cell receptor (TCR), STAT3, and NOTCH pathways establish clone-specific transcriptomic abnormalities and further accelerate their proliferative potential to develop highly malignant clones, leading to disease onset and progression [72]. According to this report, the CD45RO^+^ CD25^+^ CD7^−^ CD194^+^ Treg fraction was highly correlated with HTLV-1 proviral load in ACs, which could be attributed to a clonal expansion of HTLV-1 infected cells with additional phenotypic alterations during transformation. CD62L mRNA was increased in ATL tumor cells contrary to its decreased cell surface expression. This finding seems to be consistent with in vivo dynamics of acute type ATL cells recirculating between secondary lymphoid organs, lymph and blood. Another understated but important finding was that CD4^+^ CD45RA^+^ CD7^+^ CD62L^+^ naïve T cell fraction contained some definite *HBZ* mRNA positive HTLV-1 infected cells, which demonstrate that HTLV-1 infected naïve T cells were present in blood in ACs, presumably recirculating as normal naïve T cells.

The genetic process of ATL development from non-malignant HTLV-1-infected T cells was individually monitored by chronological studies with advanced genomic sequencing. In 2020, A. G. Rowan et al. reported that pre-malignant cells bearing putative driver mutations were detectable in the peripheral blood prior to overt ATL during 10 years of clinical observation [73]. In 3 of 6 cases, transformation was temporally linked to detection of a new cluster of mutations, at least one of which was a known ATL-driver, indicating that HTLV-1 infected cells containing known oncogenic driver mutations can persist stably for many years, before additional mutations cause progression to ATL. In 2021, M. Yamagishi et al. analyzed samples of 10 HTLV-1 carriers including four with ATL progression over nearly 10 years [74]. Several somatic mutations in key genes were detected in all ACs regardless of the disease onset and viral clonality. Recurrent mutations in TCR pathway genes (PLCG1, VAV1, PRKCB, or CARD11) were detected in 4 cases of asymptomatic HTLV-1 carriers who developed ATL later. Multiple mutations in the TCR, STAT3, and NOTCH pathways were associated with the accumulation of transcriptomic abnormalities in founder clones. The enhanced proliferative potential was commonly detected in evolved clones with different mutation patterns. These two chronological studies clearly demonstrate that accumulation of the driver gene mutations played key roles in final stage of long-term evolution of HTLV-1-infected cells.

To summarize what has been pointed out so far: (1) HTLV-1 infected CD7^+^ CD45RA^+^ naïve T cells are present in peripheral blood in ACs, (2) some of them appear to transform to premalignant Treg like phenotype clones step-by-step along with accumulation of genetic aberrations without cognate antigen stimulation, (3) a malignant clone with growth advantage becomes dominant after clonal selection. What is stated here is that HTLV-1 infection can induce development of ATL by increasing the incidence of driver gene mutations, while HTLV-1 infected T cells without such mutations stably remain non-malignant.

### Putative precursors of ATL cells

Epidemiological evidence has indicated that HTLV-1 infection during childhood and therefore mother-to-child transmission is the most important determinant of overall ATL incidence [75]. This observation has attracted attention as getting to the core of the matter but has lacked hitherto appropriate reasoning. As I wrote in Background, it is one of the fundamental issues in ATL oncogenesis and can be paraphrased as why HTLV-1 infection of neonatal naïve T cells is the most critical event throughout the course of ATL oncogenesis. If the inception of ATL is traced back to this early event, development of ATL can be interpreted as a long term fate of HTLV-1 infected naïve T cells, which seems to be the clue to understand the nature of ATL.

Recent progress in basic immunology has disclosed the distinct nature of neonatal T cells [76]. Neonatal naïve T cells are exceptionally long-living, actively maintained in a quiescent state, and have a propensity to differentiate into Treg cells as compared to adult naïve T cells. Their quiescence is characterized by the absence of LINE1 expression while LINE1 is overexpressed in aged T cells, inducing a concomitant decrease in the protein synthesis rate and contributing to the immunosenescent phenotype [77].

Little is known about the cell fate of neonatal naïve T cells after mother-to-child HTLV-1 infection during childhood. However, a case report of polyclonal expansion of HTLV-1-infected cord blood-derived T cells after cord blood transplantation (CBT) in an acute type ATL patient has offered a glimpse of how these cells remain and behave in vivo in an allogeneic situation [78]. According to this report, polyclonal expansion of CD4^+^ CD7^+^ CADM1^+^ T cells was observed 6 months and 1.5 years after CBT but was controlled after immunosuppressive treatment. At around 4 years after CBT, the patient had no relapse of ATL nor had developed chronic GVHD but retained the status of HTLV-1 carrier. These findings suggest that HTLV-1-infected neonatal T cells in vivo are phenotypically similar to those previously described in adult HTLV-I ACs and that HTLV-1-induced transient proliferation of these cells does not directly lead to development of ATL. If HTLV-1-infected neonatal naïve T cells are long-living as uninfected normal counterparts, we can speculate that HBZ^+^ CD4^+^ CD45RA^+^ CD7^+^ CD62L^+^ T cells detected in blood of ACs were the offspring of HTLV-1 infected neonatal naïve T cells or Tscm derived from them.

The gradual decrease of CD7 and the stepwise phenotypic change toward Treg like cells along with accumulation of driver gene mutations in ACs implies that the direct precursors of ATL cells were HTLV-1 infected CD7^+^ CD45RA^+^ CD62L^+^ naïve T cells or their derivative Tscm which had not experienced exogenous cognate antigen stimulation. This assumption is consistent with the recent report indicating that the number of peaks of the category of Quiescent/Low in ATL CD4^+^ T cells was high compared to that in healthy CD4^+^ T cells in assay for transposase-accessible chromatin with high-throughput sequencing (ATAC-seq) [79, 80]. The typical immunophenotype of acute type ATL cells, CD3^+^ CD4^+^ CD25^+^, CCR4^+^, CD7^lo^, and FOXP3^+^, is deceiving in case the issue of the cell of origin is discussed, because HTLV-1 infection and subsequent genetic mutations can mimic cognate antigen stimulation and accessory signals to induce distorted differentiation to effector memory T cell like phenotype. The comparative transcriptome analyses with other types of PTCL and high expression of CD62L mRNA in ATL cells suggest that this change of phenotype is not accompanied by substantial differentiation in its entirety to either type of effector Th cells. In line with this, Y. Nagai et al. demonstrated in 2015 that a minor population of CD4^+^ CCR7^+^ CD45RA^+^ CD45RO^–^ CD95^+^ CD122^dim^ Tscm-like cells were present in peripheral blood of ATL patients and only this population could repopulate identical ATL clones efficiently in immunodeficient mice [81].

### Conceivable scenario of ATL development

**Fig. 1:**
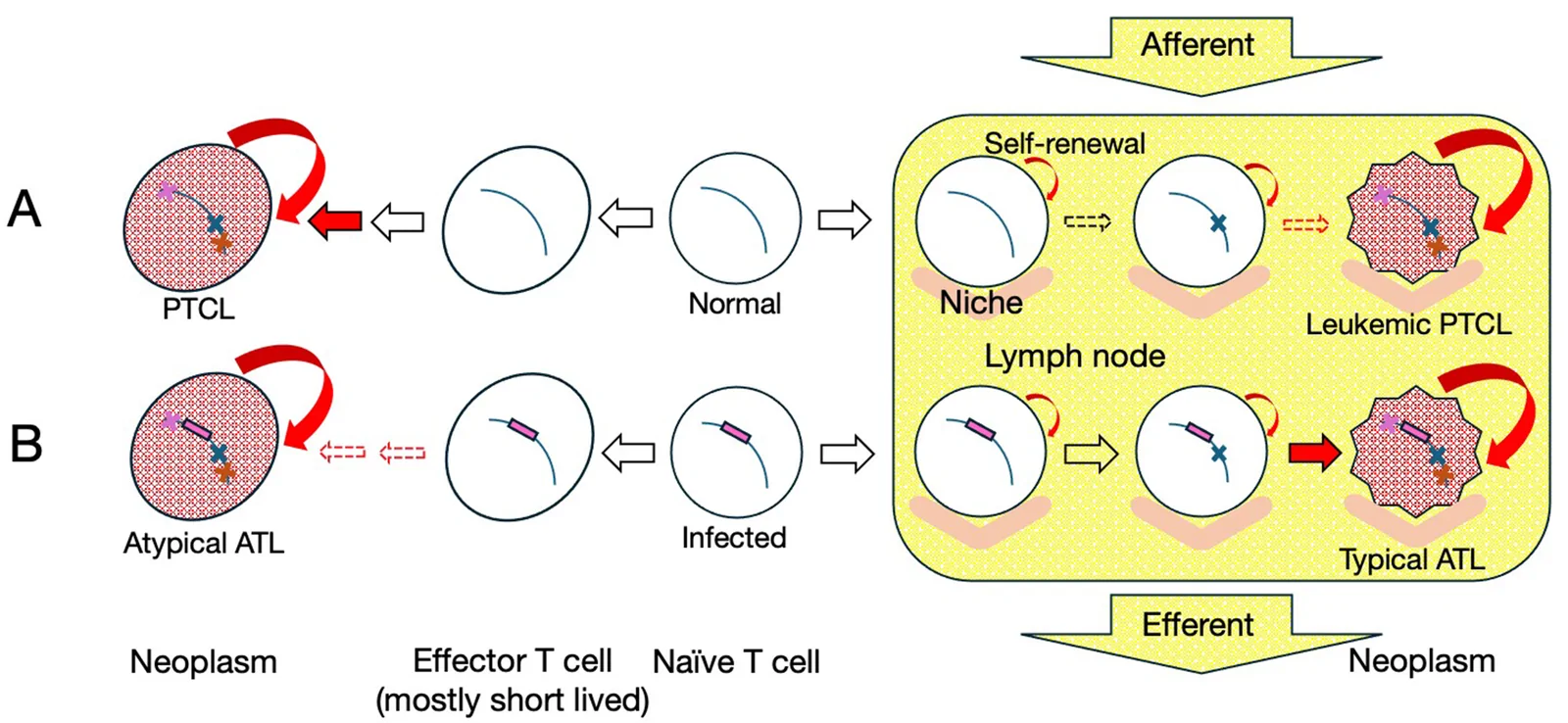
Long-term fate of neonatal naïve T cells after HTLV-1 infection. Naïve T cells and their derivative Tscm continuously recirculate between secondary lymphoid organs through afferent and efferent lymphatic vessels, lymph and blood. In lymph nodes, they repeat self-renewal in niche in quiescence. Upon antigen stimulation, they differentiate into effector T cells and then effector memory T cells, most of which are relatively short-lived. Step wise accumulation of driver gene mutations indicated by x in naïve T cells or Tscm leads to development of typical ATL or leukemic PTCS such as subtypes of T-PLL and Sézary syndrome. HTLV-1 infected effector T cells can be transformed to generate lymphoma (atypical ATL) through similar process as HTLV-1 non-infected counterparts (PTCL). A, The fate of normal neonatal naïve T cells. B, The fate of HTLV-1 infected neonatal naïve T cells. The presence of HTLV-1 provirus is indicated by a small red rectangle

Based on these previous research findings, I would like to present a scenario of ATL development as illustrated in Fig. 1. During the initial phase, neonatal naïve T cells react to HTLV-1 infection with transient expansion as well as some phenotypical changes but remain basically as naïve T cells because this is not the cognate antigen stimulation accompanied by polarization toward either type of helper T cell. HTLV-1 infected T cells could recognize their cognate antigens presented by antigen presenting cells and differentiate into effector T cells, which might evoke aberrant immune reactions as observed in HTLV-1 associated inflammatory disorders. For example, HTLV-1 infected Th1-like effector T cells have been detected in the cerebrospinal fluid and spinal cord lesions of HTLV-1–associated myelopathy/tropical spastic paraparesis (HAM/TSP) [82].

Such HTLV-1 infected effector T cells seem to have been eliminated or suppressed in most ACs. It is possible that exogenous cognate antigen stimulation could break the latency to render HTLV-1 infected cells vulnerable to CTL or unsuitable for niche in secondary lymphoid organs. The immune suppression may be ascribed to the nature of neonatal naïve T cells before HTLV-1 infection, a significant part of which consist of Treg cells [55, 83] or the effects of HTLV-1 HBZ on infected T cells themselves and its secondary effects on surrounding cells in the microenvironment toward tolerant state [84, 85].

As time passes, most of HTLV-1 infected effector or effector memory T cells cease to exist. In contrast, offspring of the HTLV-1 infected neonatal naïve T cells survive as naïve T cells or their derivative Tscm in niche in lymph nodes, recirculating in blood and lymph for decades. These cells require weak but steady growth signals from the microenvironment of niche for self-renewal with asymmetric cell division like other types of stem cells to generate Tscm [52, 86]. ATL cells in lymph nodes have been reported to display higher expression of the Ki-67 antigen than ATL cells in peripheral blood [87], suggesting that proliferation of ATL cells is still dependent on niche in lymph nodes. Normal peripheral naïve T cells have been demonstrated to increase the frequency of cell division after the supply of central naïve T cells is disrupted [88–90]. In other words, middle-aged and older people have more self-renewing naïve T cells and their derivative Tscm in the periphery than younger people and, accordingly, have higher probability of genetic mutations and chromosomal abnormalities. If HTLV-1 infected naïve T cells behave in the same way, this may explain the sharp increase of incidence of ATL in the middle age [14].

What has been said so far corresponds to typical ATL. It is possible that HTLV-1 infected effector T cells survive in rare cases and develop into atypical nodular or cutaneous types of ATL which are similar to angioimmunoblastic T cell lymphoma, anaplastic large cell lymphoma, or mycosis fungoides through accumulation of genetic changes. Conversely, on infrequent occasions, normal naïve T cells or their derivative Tscm can transform without HTLV-1 into leukemic PTCL such as Sézary syndrome and T-PLL. Although the cell of origin of these leukemic PTCL is under investigation, circulating malignant cells in both have been reported to be of naïve T cell phenotype at least in some cases [91, 92].

### Driver gene mutations

Whole genome sequencing studies have revealed many genetic aberrations in ATL, most of which were shared across other subtypes of PTCL [46, 93–95]. Mutations in genes related to T cell activation signaling were recurrently detected in mature T cell neoplasms in general. Among those, molecules involved in TCR signaling and NF-κB pathway such as STAT3, PLCG1, CARD11, PRKCB, and IRF4 were most frequently altered, and high incidence of deletions at the NRXN3 locus and mutually exclusive alterations in CIC or ATXN1 was noted in ATL [96]. It is unlikely that all the numerous mutations detected in ATL samples are required for the onset of the disease but a few or at least multiple mutations seem to be involved in the stepwise development of ATL, which can be explained by the well-known theory on the underlying mechanism of T cell proliferation. Accumulating evidence has indicated that TCR stimulation and other accessory signals of costimulation and cytokines converge on an increase in a certain factor such as Myc or mTORC1, which determines the duration and cycles of cell division in T cells [97–100]. More recent reports have suggested that the NAD^+^ in the end controls a quiescence exit checkpoint and rate of cell cycle entry [101, 102]. The T cell activation system is equipped with the multiplex feedback or fail-safe networks to prevent excessive signals [103, 104]. Therefore, gain-of-function and/or loss-of-function genetic aberrancies in this system can cause neoplastic growth of T cells, if the net amount of the factors, Myc, mTORC1, or NAD+, exceeds the threshold regardless of difference in combination of mutated genes. This seems to be the reason why such multiple but a different set of recurrently mutated genes related to T cell activation signaling are detected in ATL and other types of PTCL. In the case of HTLV-1 infected cells, the provirus genome having the CTCF binding site [105] and the viral enhancer [106] together with the viral factors should add some extra to the fore-mentioned net amount of the factors through epigenetic as well as genetic processes.

It is known that HTLV-1 infection imposes high risk of genetic alterations on T cells through induction of APOBEC3A and/or related enzymes and acceleration of cell growth [107–110]. The former is considered to be a part of innate immune responses through triggering of their own pattern recognition receptors (PRRs) such as Toll like receptors (TLRs), mitochondrial antiviral signaling protein (MAVS) and retinoic acid-inducible gene I-like receptors (RLRs) in T cells [111, 112], which must have deep and prolonged influences on their subsequent fate particularly in the case of latent infection. Thus, in my view, there is another mechanism of genetic risk in HTLV-1 infected T cells, namely, extra transcription-replication conflicts resulting from the innate immune response of their own. Evidence has indicated that open chromosomal domains with gene expression have higher genetic vulnerability because the transcription poses a roadblock to replication, fork progression, promoting replication stress [113–115]. NRXN3 was shown to be highly expressed in ATL cells and in *HBZ* transgenic mice [116]. As to CIC, a recent report has indicated that its expression is induced upon viral infection [117]. Taken together, these genetic alterations frequently detected in ATL cells may be ascribed to HTLV-1 infection.

### Relationship between ATL and HTLV-1

After discovery of HTLV-1 in 1980, ATL was redefined as a T cell neoplasm caused by HTLV-1 in 1984. Ever since, extensive studies have revealed unique properties of HTLV-1 related to ATL but failed to provide decisive evidence that ATL is directly caused by HTLV-1. In the 2010s and early 2020s, whole genome sequencing studies detected many genetic aberrations in ATL, most of which were shared across other subtypes of PTCL, suggesting that ATL is caused by accumulation of genetic alterations as other cancers in general. Despite these new findings, the etiological concept of ATL has not been revisited and diagnosis of ATL has been practically based on detection of monoclonally integrated HTLV-1 provirus in tumor cells until today. Numerous review papers on ATL have been published as ever based on the preconceived notion that HTLV-1 viral factors play central roles in its oncogenesis. While recent reviews have incorporated the genetic aberrations as supplementary elements to viral factors [118–120], they have not paid attention to the developmental aspect of HTLV-1 infected T cells and left most of the fundamental questions unaddressed. In fact, no understandable explanation has been given to why HTLV-1 does not cause ATL in ACs.

Any type of cancer initiates from a somatic cell capable of self-renewal when it undergoes genetic aberrations that cause either differentiation defects and/or sustained proliferation. These changes must be compatible with the differentiation stage of the cell, the microenvironment/niche, and the nature of the tissue [121]. Cancers associated with oncogenic viruses, even those with active oncogenes such as v-src of Rous sarcoma virus, are no exception to this, and viral factors alone are not sufficient to cause cancer [122]. Human oncogenic viruses including HTLV-1 and EBV share common capability to establish latent chronic infections, during which cellular gene mutations can be induced and accumulated ultimately leading to cancer [123].

The causality of HTLV-1 to ATL is similar to that of other oncogenic viruses to cancers. The relationship between Burkitt lymphoma and EBV provides a useful precedent to sort out the relationship between ATL and HTLV-1. Both viruses can activate normal lymphocytes and induce polyclonal expansion but not directly cause leukemia/lymphoma by themselves. The endemic type Burkitt lymphoma was once thought to be caused by EBV but now EBV is not included in the definition of the disease. Burkitt lymphoma is currently understood as a lymphoma derived from germinal center B cells involving dysregulation of MYC expression, whether it is associated with EBV infection or not. Likewise, the etiological definition of ATL might need rethinking.

## Conclusions

Combining information from multiple sources of epidemiologic, pathologic, immunologic and genomic studies, it is possible that the cell of origin of ATL is a neonatal naïve T cell that experienced HTLV-1 infection during early childhood. HTLV-1 infected neonatal naïve T cells or their derivative Tscm appear to be long-living, actively maintained in a quiescent state in niche, and have a propensity to differentiate into Treg cells as uninfected normal counterparts. Normal naïve T cells and Tscm slowly cycling in quiescence are supposed to have much less risk of genetic mutations compared to effector T cells that experience antigen stimulation, clonal expansion and differentiation into either type of effector Th subsets. Latent infection of HTLV-1 is mediated by not only HBZ but also by the silencing element in the provirus and the host factor RUNX1 [124]. Such quiescence state and the viral complete latency may have allowed infected naïve T cells to evade genetic mutations as well as immune surveillance for decades. However, the presence of provirus affects their long-term fate not only by the viral factors and genome but also through signaling of their own PRRs. When these cells accelerate the rate of cell division as the supply of central naïve T cells decreases in the middle age, the risk of genetic aberrations gets higher than normal counterparts due to induction of APOBEC3 and related enzymes, promotion of cell growth, and PRRs-mediated extra transcriptional overload.

In sum, the distinctive feature of ATL lies in its cell of origin which may involve a neonatal naïve T cell and in persistence of HTLV-1 provirus that facilitates genetic aberrations. The deductive reasoning can not only answer all the fundamental questions but also explain the reason why mutations in NRXN3 and CIC are frequently detected in ATL. These assumptions, however, should be proven by actual human specimens. The primary limitation is lack of information regarding the status of naïve T cells during early phase of mother-to-child HTLV-1 infection. A 10-year follow up of vertically HTLV-1 infected children has been reported but only to monitor changes in proviral loads and clonality [125]. Further studies are required to fully characterize HTLV-1 infected neonatal naïve T cells and their derivative Tscm from infancy to each age stage in ACs. As to diagnosis and treatment of ATL, the driver gene mutations causing each ATL case should be individually identified for risk prediction and future molecular target therapy, despite expected difficulty due to the multiplicity and diversity. Obviously, the top priority is the prevention of mother-to-child HTLV-1 infection, and for ACs, discovery of novel concept drugs such as molecular glues [126] to HBZ or paradoxically to RUNX1 is awaited to minimize HTLV-1 infected premalignant naïve T cells.

## Data Availability

No datasets were generated or analysed during the current study.

## Abbreviations

ACs: Asymptomatic carriers
ATAC-seq: Assay for transposase-accessible chromatin with high-throughput sequencing
ATL: Adult T cell leukemia
CBT: Cord blood transplantation
EBV: Epstein-Barr virus
FFPE: Formalin-fixed paraffin-embedded
HAM/TSP: HTLV-1–associated myelopathy/tropical spastic paraparesis
HBZ: HTLV-1 bZIP factor
HTLV-1: Human T cell leukemia virus type 1
MAVS: Mitochondrial antiviral signaling protein
PRRs: Pattern recognition receptors
PTCL: Peripheral T cell lymphomas
RLRs: Retinoic acid-inducible gene I-like receptors
TCR: T cell receptor
Th: Helper T cell
TLRs: Toll like receptors
T-PLL: T prolymphocytic leukemia
Tscm: Stem cell-like memory T cells
